# Long-term artificial selection of Hanwoo (Korean) cattle left genetic signatures for the breeding traits and has altered the genomic structure

**DOI:** 10.1038/s41598-022-09425-0

**Published:** 2022-04-19

**Authors:** Dongwon Seo, Doo Ho Lee, Shil Jin, Jung Il Won, Dajeong Lim, Mina Park, Tae Hun Kim, Hak Kyo Lee, Sidong Kim, Inchul Choi, Jun Heon Lee, Cedric Gondro, Seung Hwan Lee

**Affiliations:** 1 Division of Animal and Dairy Science, Chungnam National University, 99, Daehak-ro, Yuseong-gu, Daejeon, 34134 South Korea; 2 Hanwoo Research Institute, National Institute of Animal Science, RDA, Pyeongchang, South Korea; 3 Animal Genomics and Bioinformatics Division, National Institute of Animal Science, RDA, Jeonju, Korea; 4 Animal Breeding and Genetics Division, National Institute of Animal Science, RDA, Seonghwan, South Korea; 5 Poultry Institute, National Institute of Animal Science, RDA, Pyeongchang, South Korea; 6 Department of Animal Biotechnology, Jeonbuk National University, Jeonju, South Korea; 7 Beacon Center for the Study of Evolution in Action and Department of Animal Science, Michigan State University, East Lansing, USA

**Keywords:** Evolution, Genetics

## Abstract

Indigenous Korean breeds such as Hanwoo (Korean) cattle have adapted to their local environment during the past 5000 years. In the 1980s, the National Genetic Improvement Program was established to develop a modern economic breed for beef production in Korea through artificial selection. This process is thought to have altered the genomic structure of breeding traits over time. The detection of genetic variants under selection could help to elucidate the genetic mechanism of artificial selection in modern cattle breeds. Indigenous Hanwoo cattle have adapted in response to local natural and artificial selection during a 40-year breeding program. We analyzed genomic changes in the selection signatures of an unselected population (USP; n = 362) and a selected population (KPN; n = 667) of Hanwoo cattle. Genomic changes due to long-term artificial selection were identified using a genome-wide integrated haplotype score (iHS) and a genome-wide association study (GWAS). Signatures of recent selection were detected as positive (*p*iHS > 6) or negative (*p*iHS < –6) iHS scores spanning more than 46 related genes in KPN cattle, but none in USP cattle. A region adjacent to the *PLAG1* gene was found to be under strong selection for carcass weight. The GWAS results also showed a selection signature on BTA14, but none on BTA13. Pathway and quantitative trait locus analysis results identified candidate genes related to energy metabolism, feed efficiency, and reproductive traits in Hanwoo cattle. Strong selection significantly altered Hanwoo cattle genome structural properties such as linkage disequilibrium (LD) and haplotypes through causal mutation for target traits. Haplotype changes of genome structure which are changes of ancestral allele to derived alleles due to selection were clearly identified on BTA13 and BTA14; however, the structure of the LD block was not clearly observed except BTA14. Thus, selection based on EBVs would be working very well in Hanwoo cattle breeding program appears to have been highly successful.

## Introduction

Livestock animals domesticated about 10,000 years ago^1^, followed by subsequent breed formation through artificial selection, which caused significant changes in livestock genomes^2^. Hanwoo (Korean) cattle have subsequently undergone modern breed formation to develop very small-framed taurine cattle over the past 5000 years on the Korean Peninsula. During this period, Hanwoo cattle have been used extensively for farming and transportation; over time, they have evolved to be a meat-producing breed. In the 1980s, a breeding program was established for full-scale Hanwoo production for meat^3^, resulting in a yearling weight (YW) of 0.78 kg/year, carcass weight (CWT) of 0.35 kg/year, and eye muscle area (EMA) of 0.27 cm^2^/year^3^. The annual genetic gain of the Hanwoo breed has continually increased within the breeding program, due to strong selective pressure on breeding objective traits^3^. The detection of single-nucleotide polymorphisms (SNPs) on selection loci can be used to reveal genetic mechanisms that have major effects on genetic differences between selected and unselected populations; however, identifying the proper methodology for selection signature identification depends on several factors, such as the genetic similarity of the founder animals and recent selection events^2^. The development of analytical methods for genomic data using dense SNP markers^4^ has facilitated the quantification of selection effects within the entire cattle genome; these methods include genome-wide association study (GWAS)^5^ and/or selection signature analysis^6^ approaches. Both selection signature analysis and GWAS exploit linkage disequilibrium (LD) that form in haplotype structure due to natural and artificial selection over long periods. Both methods have been used successfully to identify genomic changes due to selection in livestock populations^7–9^ and are therefore appropriate for identifying similar genomic regions that have undergone strong selection in cattle breeding programs. However, unmatched results can also occur due to potential bias in GWAS^10^. GWAS was applied to identify significant loci for carcass traits in small experimental Hanwoo steer samples^11^, and identified ubiquitous genomic regions including *PLAG1* and *CHCHD7* (23 Mb) on BTA14. Besides, Cattle QTLdb (https://www.animalgenome.org/cgi-bin/QTLdb/BT/index) analyzed 1030 papers from 1994 to 2020, reporting 160,659 QTL and association areas for 675 different traits, including cow economic traits. This information can help infer relevant features for the selection signal’s genomic regions. Among the contents reported in QTLdb, Huson et al. reported the results consistent with the signature of selection and GWAS results for the slick-hair coat (SLICK) of Senepol cattle in Spain. Bahbahani et al. also reported haplotypes for milk production, immunity, breeding, and heat resistance QTLs based on signature of selection and GWAS analysis results of two kinds of Zebu cattle breeds, Butana and Kenana, from Sudan^12,13^.

In this study, we performed genome-wide analyses to detect genomic regions undergoing recent strong selection using an integrated haplotype score (iHS) to identify derived alleles based on ancestral alleles associated with breeding traits in selected (KPN) and unselected (USP) Hanwoo populations. We then compared extended haplotype homozygosity (EHH) values and changes in the estimated breeding values (EBVs) of breeding animals that were selected at 10-year intervals in a KPN breeding population to investigate whether EHH values reflect genetic improvement in Hanwoo cattle. Finally, to determine whether the mutations within significant genome regions included the causal mutation for breeding objective traits, we validated the selection signature loci using a functional annotation such as GO and gene enrichment analyses.

## Materials and methods

### Ethical statement

The experimental cattle blood sample collection and genotyping procedures followed the standards established by the Committee for Accreditation of Laboratory Animal Care at National Institute of Animal Science (NIAS) in Korea. The institutional Animal Care and Use Committee (IACUC) of NIAS, RDA approved this experiment (permit No. 2017-251). This study complied with the ARRIVE guidelines.

### Animals and genotypes quality control

**Figure 1:**
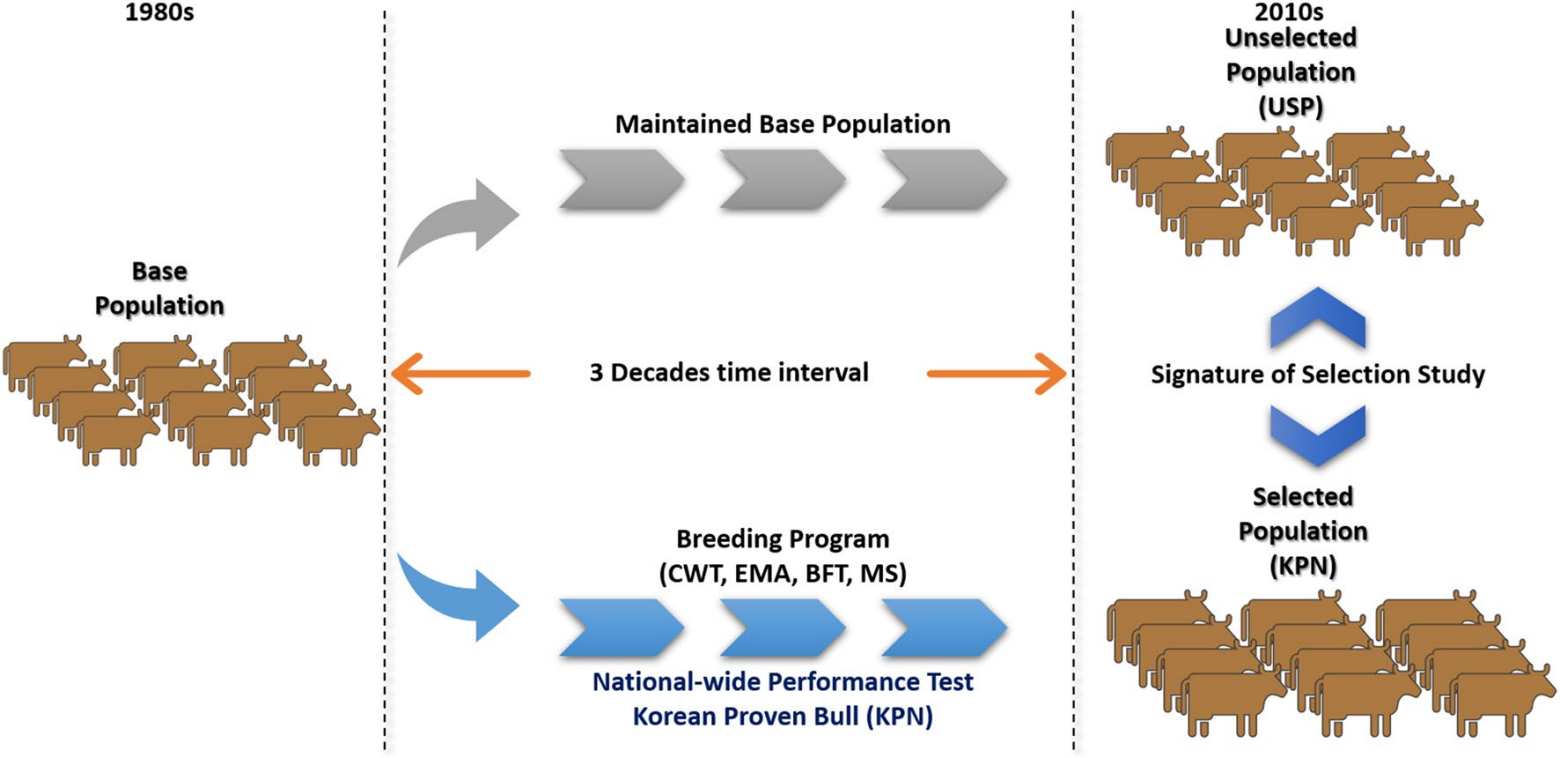
Schematic diagram of selected (KPN) and unselected (USP) Hanwoo cattle populations in Korea.

We used DNA samples from a total of 1063 Hanwoo cattle, divided into a selected population (KPN; n = 667) which have undergone strong artificial selection during the past 30 years as a result of breeding program and an unselected population (USP; n = 362) which was maintained for research and conservation at the Hanwoo Research Institute in National Institute of Animal Science (Fig. 1). KPN cattle are a seed stock population of proven bulls selected using performance and progeny testing at the Hanwoo Improvement Center of the National Agricultural Cooperative Federation in Seosan, Chungnam Province, Korea. USP cattle are a base population that has maintained its initial genetic composition for research and conservation at the Hanwoo Research Institute of the National Institute of Animal Science, RDA, Korea. All samples were genotyped for 54,609 SNPs using an Illumina BovineSNP50 BeadChip ver. 2 array (Illumina, San Diego, CA, USA). The quality of genome-wide data was maintained through SNP filtering using PLINK ver. 1.9 software^14^; we removed SNPs that were out of Hardy–Weinberg Equilibrium (P < 10^–4^) and had a low call rate (< 90%) or high rate of missing genotypes (> 10%). To reduce bias in the data, we limited minimum minor allele frequency (MAF) to 1%. After genotype data cleaning, the remaining SNPs were used for further analysis, i.e., 43,834 (79.3%) and 42,936 (77.2%) SNPs on the 29 autosomal chromosomes of the KPN and USP Hanwoo populations, respectively. We used the *ARS-UCD1.2* whole-genome assembly of *Bos taurus* to obtain the genome coordinates of all available SNPs for selection signature analysis.

### Population structure analysis

A four-dimensional pairwise genetic distance matrix was calculated using a multi-dimensional scatter (MDS) plot algorithm in PLINK ver. 1.9 software^14^ and expressed as coordinates in R software (R Core Team 2015). We used Admixture ver1.3 software^15^ to detect admixture of the populations and predict their relationships using ancestor model adjustment or k-means clustering. To maintain balanced sample numbers between the populations for the MDS and admixture analyses, we used all KPN and USP samples. The fixation index (*F*_*st*_) was calculated for the measure of population differentiation due to genetic distance, as follows:

$${F}_{st}= \frac{{H}_{T}-{H}_{S}}{{H}_{T}}$$

Of this, H_T_ is expected heterozygosities for total population and H_S_ is expected heterozygosities in sub-population.

### Signature of selection analysis and GWAS

We used iHS (integrated Haplotype Score) test to identify selection signatures in Hanwoo cattle^9,16^. It is designed to detect regions with a high level of haplotype homozygosity over an unexpected long-distance (relative to neutral expectations). The iHS scores were calculated through the package ‘*rehh*’ as the natural log ratio of integrated EHH (integrated extended haplotype homozygosity; iHH) using a long-range haplotype-based method between ancestral and derived alleles for each SNP within each *Bos taurus* population^17^. Allele status information (i.e. ancestral/derived) was established from previous *Bos taurus* cattle studies^2,18^. The iHS statistic is obtained by detecting regions that are rich in haplotype homozygosity using only natural logarithm ratios of the iHS of ancestral and derived alleles on each SNP with MAF > 0.01. The negative iHS values indicate extended haplotypes of derived alleles by recent selection and positive iHS scores can be detected as ancestral alleles by unintended economic traits or the genetic hitch-hiking effect. Therefore, these positions can be used to detect genomic regions under selection. Using fastPHASE 1.4.0 software^19^, we identified phased haplotypes among all autosomes in both cattle populations, using adjusted options as previously described Gautier and Naves^6^. This analysis approach was based on within- and between-population analysis methods. Within-population analysis was performed by integrating EHH for ancestral (iHHa) and derived (iHHd) alleles. EHH decreases as the distance from a core SNP increases. We define iHS as the ratio of iHHa to iHHd, as follows:

$$iHS = {\text{log}}\left( {{{iHH_{a} } \mathord{\left/ {\vphantom {{iHH_{a} } {iHH_{d} }}} \right. \kern-\nulldelimiterspace} {iHH_{d} }}} \right).$$

Since iHS generally follows the normal distribution, we used MAF as the basis for the mean and variance of these values and standardized the distribution to a mean of 0 and a standard deviation of 1. Thus, ancestral and derived alleles have positive and negative values, respectively. For an easier understanding of positive and negative iHS values in each chromosome where the signature of selection, we transformed it to piHS values. The iHS value is constructed to have an approximately standard Gaussian distribution which can compare between markers is possible without being affected by allele frequency. Therefore, the p-value for positive and negative iHS values can be calculated by applying the Gaussian cumulative density function, as follows:

$$piHS= -log10(1-2\left|\phi \left(iHS\right)\right.-\left.0.5\right|).$$

Of this, statistically significant SNPs were set to piHS > 6 for ancestral and derived alleles of signature of selection.

The Rsb (Ratio of EHH between populations) distribution used median and standard deviation of Rsb; however, we used a different standardization procedure than that used for iHS by Tang et al.^20^; this procedure uses the median instead of the mean to decrease the impact on extreme data points without dividing the score by a binary.

$$Rsb=log\left(\frac{{iES}_{pop1}^{Tang}}{{iES}_{pop2}^{Tang}}\right).$$

Positive and negative values imply selection for the populations used in the numerator and denominator, respectively. We derived the p-value as follows:

$${P}_{Rsb}= -{log}_{10}(1-2\left|\phi \left(Rsb\right)-0.5\right|)$$

The carried out iHS, Rsb values followed a Gaussian distribution. Therefore, we used bifurcation plots to evaluate the selection footprints based on the positions of LD blocks with increasing distance from selected SNPs that were strong candidates within populations (iHS) and between populations (Rsb).

To identify changes in the haplotype pattern and LD block in the selection signal area, the LD structure of each population was identified using the Haploview software from Broad Institute^21^. LD structure was confirmed the significant SNP position of the selection signal and adjacent SNPs lists generated Haploview format through the recode option in plink1.9 software and applied the Linkage format default option (ignore pairwise parallels of markers > 500 kb with > 50% missing).

We performed GWAS for the KPN population. To test the association between SNPs and causal mutations under selection, we used the estimated breeding value (EBV) as high-accuracy (> 80%) phenotype information on carcass traits, including carcass weight (CWT), backfat thickness (BFT), marbling score (MS), and eye muscle area (EMA). Markers were assumed to be in LD with quantitative trait loci (QTL) in close proximity; this effect was found to be an additive effect (QTL allele substitution effect). The additive effect of an SNP on each phenotype was fitted using regression analysis, with values in the covariate coded as 0, 1, or 2 copies of the variant allele after fitting the following mixed-linear model^22,23^:

$$y=a+bx+{g}^{-}+e$$

where $y$ is the EBV, $a$ is the overall mean, $b$ is an additive fixed effect, $x$ is the genotype matrix, ${g}^{-}$ is the accumulated effect of all SNPs, except for the cumulative effect on candidate chromosome SNPs as a random effect, which is random error. The variance ${g}^{-}$ was re-estimated every time a chromosome was excluded during genetic relationship matrices (GRM) calculation. The estimation of the genetic relationship from genome-wide SNPs were calculated as follows^22^:

$${A}_{jk}=\frac{1}{N}\sum_{i=1}^{N}\frac{({x}_{ij}-2{p}_{i})({x}_{ik}-2{p}_{i})}{2{p}_{i}(1-{p}_{i})}$$

where ${A}_{jk}$ is the genetic relationship between individuals *j* and *k* (GRM), ${x}_{ij}$ is the number of copies of the reference allele for the *i*th SNP of the *j*th individual, ${x}_{ik}$ is the number of copies of the reference allele for the *i*th SNP of the *k*th individual, and ${p}_{i}$ is the frequency of the reference allele. We used an option of MLM leaving-one-chromosome-out (LOCO) analysis.

### The functional enrichment analysis

Gene ontology (GO) and gene pathways were analyzed to determine the functions of genes adjacent to SNP regions with significant selection signatures. In such regions, high-frequency ancestral and derived alleles were set as the primary targets. A list of genes within the 1-Mbp regions to the left and right of the SNP was created using the Ensemble Biomart tool (http://asia.ensembl.org/biomart/martview/0b8145a691e46ff83aa7d294581a1d33) and the Cytoscape clueGO package^24^. Fisher’s exact test was used to calculate the observed ratios to predicted values, and statistical significance was evaluated according to their p-values. The list of significant genes was divided into biological processes, molecular functions, and cellular components using Kyoto Encyclopedia of Genes and Genomes (KEGG) pathway analysis^25^ and the Cytoscape web tool^26,27^.

## Results

### Genetic structure of KPN and USP

**Figure 2:**
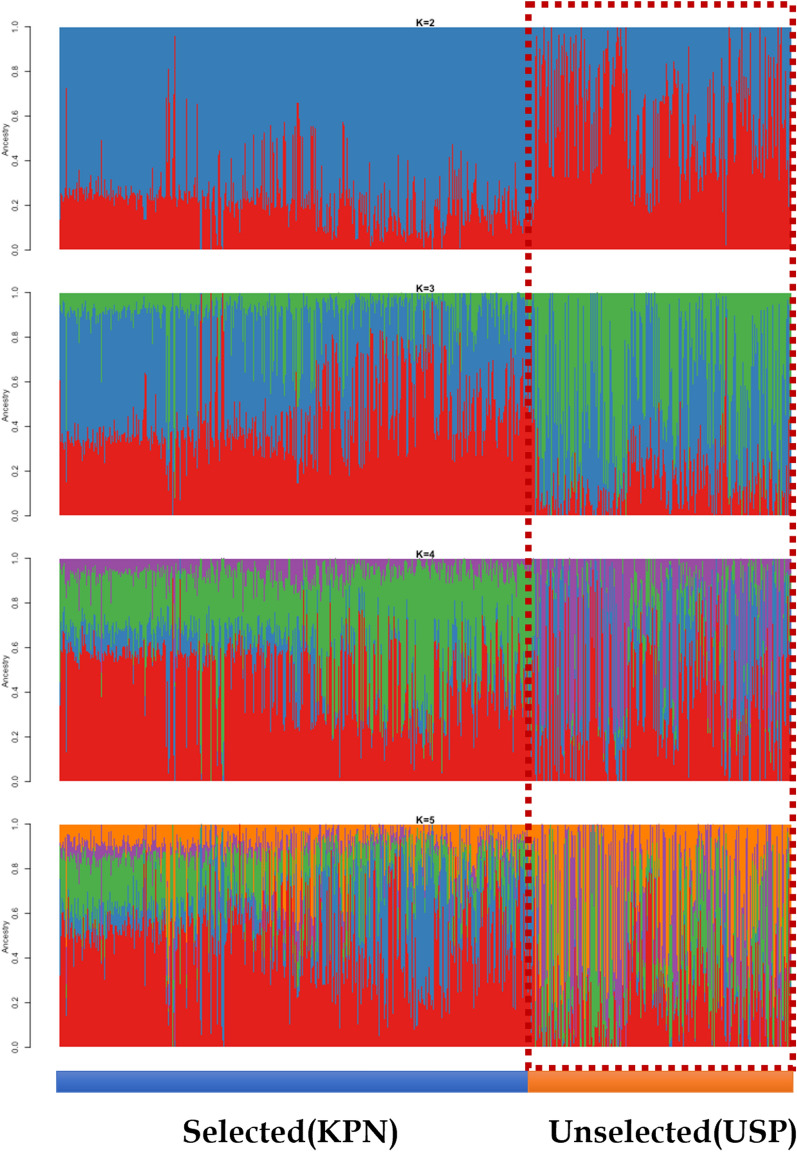
A 30-year admixture analysis of KPN and USP Hanwoo cattle. Both populations have the same genetic composition but different component ratios.

To investigate genetic differences and similarities between these populations, we performed MDS and admixture analyses (Fig. S1 and Fig. 2). The genetic components of the two populations showed both similarities and differences in their genetic composition patterns. The MDS plot showed that the KPN and USP populations were slightly different (Fig. S1), whereas the admixture analysis showed that they had different allele frequencies, despite the presence of some similar individuals (Fig. 2). The cross-validation (CV) error of the admixture analysis was 0.536, which was highest at k = 2 and decreased to 0.521 at k = 5, indicating a higher explanatory power. The fixation index (*F*_*st*_) of the KPN and USP Hanwoo populations found that the differentiation between the two groups was significantly lower at 0.0142, indicating that the two groups were sharing the genetic diversity.

### Genome-wide scan for selection signatures

Based on the standardized values of Rsb, chromosomes with a prominent selection effect in KPN over USP were identified as BTA1, 5, 10, 13, 14, 23. The area consistent with the derived and ancestral allele of iHS was on BTA13.

**Figure 3:**
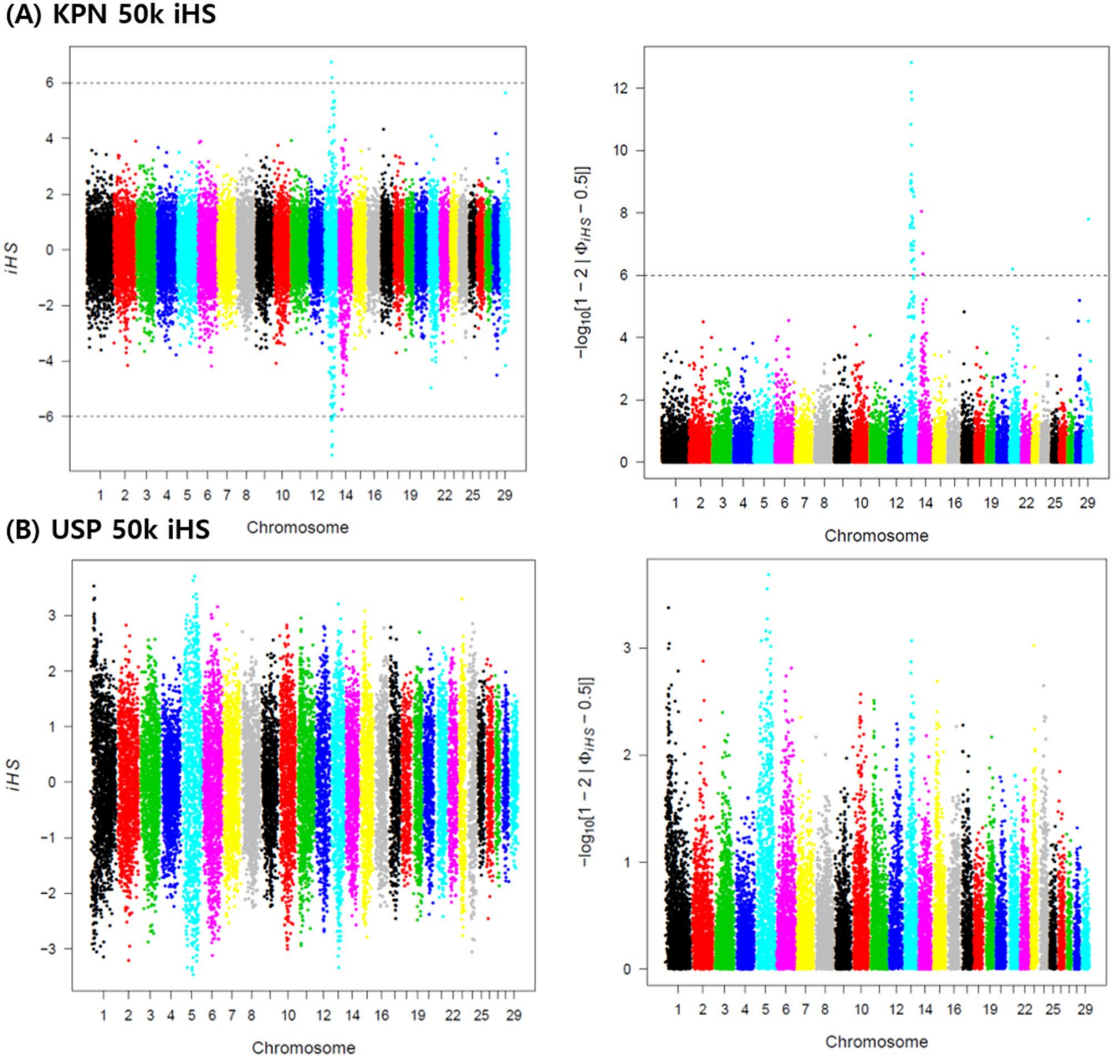
The integrated haplotype (iHS) results for selected (KPN) and unselected (USP) Hanwoo population: (**A**) is the iHS result of the KPN population and the right side is the piHS, which confirms the p-value of the calculated iHS value, (**B**) is the iHS and piHS value for USP population, respectively. The significant association of signature of selection was confirmed only KPN population.

**Figure 4:**
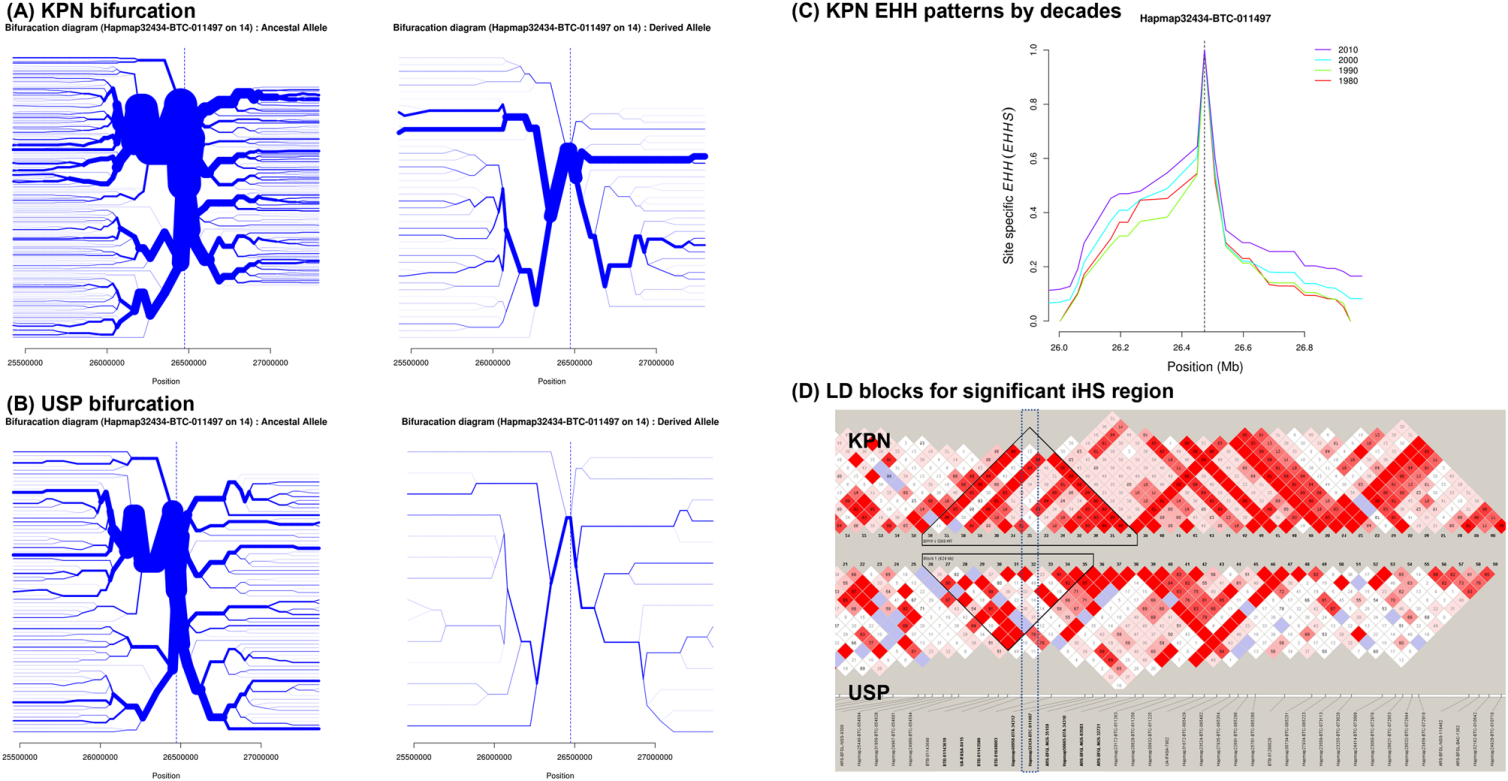
The bifurcation, EHH and Haploview results for significant derived allele in Hanwoo: (**A**) is bifurcation result of ancestral and derived allele for KPN population and (**B**) USP population. Both populations have one strong derived haplotype in this significant associated region. (**C**) EHH result for KPN population has similar pattern with ancestral allele, but 1980s and 1990s have not much differences and their (**D**) LD structure by Haploview analysis was confirmed extended block to right hand side in selected (KPN) population on BTA14 Hapmap32434-BTC-011497 SNP position. *EHH* expended haplotype homozygosity, *KPN* 30-year selected Korean proven bull, *USP* unselected population.

The calculated iHS values were normally distributed, as shown by the agreement of Gaussian distribution and QQ plots; therefore, the KPN and USP iHS values were not significantly different (Fig. S2). Statistically significant SNPs were identified on BTA13 (32), BTA14 (3), BTA21 (1), and BTA29 (1) within KPN cattle, whereas there was no significant selection signal in USP cattle (Table 1, Fig. 3). In KPN cattle, we identified 23 intergenic, 4 upstream, and 10 intron regions. SNPs in the upstream and intron regions were identified as protein-coding mutations that affect transcripts and are likely to be directly related to phenotype. The genes adjacent to significant SNPs were identified as 44 genes which detailed lists are shown in Table 1. The large negative iHS scores indicating that a derived allele and large positive iHS scores of an ancestral allele can be selected by the hitch-hiking effect. Therefore, both extreme iHS scores have the potential interest in the breeding traits^9^. We identified 29 of 37 significant SNPs as derived alleles and the remaining 8 SNPs as ancestral alleles (Table 1). Furthermore, the rs110693756 (ARS-BFGL-NGS-65199) SNP, which was in the intron of the *RIN2* gene, was identified as the most significant ancestral allele, and the rs110783267 (ARS-BFGL-NGS-14104) SNP, which is in the intergenic region between *RF00026* and *THBD*, was identified as the most significantly derived allele on BTA13 (Fig. S3). SNPs with high log-transformed iHS values (≥ 10) were all located in the intergenic region above BTA13. Three and one derived alleles were identified on BTA14 and BTA21, respectively, and only one ancestral allele was confirmed on BTA29 (Fig. 4 and Fig. S4).

**Table 1:** List of significant single-nucleotide polymorphisms (SNPs) from the integrated haplotype score (iHS) results.

Chr	Position	Marker name	rsID	iHS	− LOG_10_(*p-value*)	Gene name	Region	Allele
13	35,005,345	BTB-01342926	rs42469902	− 5.699	7.919	*ENSBTAG00000046352-JCAD*	Intergenic	G/A
13	36,785,616	BTA-32337-no-rs	rs41628271	− 5.521	7.472	*ARMC4-MKX*	Intergenic	G/A
13	37,861,193	ARS-BFGL-NGS-103989	rs109603252	− 5.578	7.613	*ENSBTAG00000006043*	Upstream	C/T
13	39,528,648	ARS-BFGL-NGS-65199	rs110693756	6.936	11.395	*RIN2*	Intron	A/G
13	40,080,019	Hapmap50934-BTA-32439	rs41628321	− 6.16	9.139	*RALGAPA2*	Intron	T/C
13	40,943,586	ARS-BFGL-NGS-22137	rs109374197	− 5.685	7.885	*PAX1-FOXA2*	Intergenic	A/T
13	41,164,136	ARS-BFGL-NGS-102025	rs110274438	− 5.421	7.227	*PAX1-FOXA2*	Intergenic	G/A
13	41,383,107	ARS-BFGL-NGS-13176	rs109765738	− 5.17	6.632	*PAX1-FOXA2*	Intergenic	G/A
13	41,433,750	Hapmap47567-BTA-32536	rs41629073	− 5.143	6.568	*PAX1-FOXA2*	Intergenic	G/A
13	41,467,021	Hapmap58859-rs29023610	rs29023610	− 5.142	6.567	*PAX1-FOXA2*	Intergenic	G/A
13	41,504,180	BTA-32538-no-rs	rs41584360	− 6.07	8.893	*PAX1-FOXA2*	Intergenic	G/A
13	41,826,984	ARS-BFGL-NGS-14104	rs110783267	− 7.64	13.662	*RF00026-THBD*	Intergenic	G/A
13	41,935,549	BTB-00523176	rs41689217	6.019	8.755	*CD93-NXT1*	Intergenic	A/G
13	42,741,708	ARS-BFGL-NGS-100409	rs110687098	− 6.898	11.278	*VSX1-PYGB*	Intergenic	G/A
13	44,040,542	BTB-01254653	rs42074017	− 6.672	10.599	*ENSBTAG00000017191-KLF6*	Intergenic	G/A
13	44,978,611	ARS-BFGL-NGS-23830	rs42496808	− 5.389	7.149	*ENSBTAG00000046128-PITRM1*	Intergenic	A/C
13	45,034,339	ARS-BFGL-NGS-18246	rs110155669	− 6.545	10.224	*ENSBTAG00000046128-PITRM1*	Intergenic	G/A
13	45,697,341	BTA-73938-no-rs	rs41590789	− 5.121	6.518	*RF00402-ADARB2*	Intergenic	G/A
13	46,089,659	ARS-BFGL-NGS-35887	rs108961732	− 5.989	8.675	*ADARB2*	Intron	G/A
13	47,725,601	ARS-BFGL-NGS-84799	rs110840150	− 5.739	8.020	*GPCPD1*	Intron	T/G
13	48,120,211	ARS-BFGL-NGS-35327	rs41696855	− 5.23	6.771	*MCM8*	Upstream	G/A
13	48,456,319	BTA-115847-no-rs	rs43706693	5.674	7.856	*FERMT1-ENSBTAG00000046562*	Intergenic	A/C
13	52,247,494	ARS-BFGL-NGS-35859	rs109671123	5.047	6.349	*PTPRA*	Intron	G/T
13	52,480,216	ARS-BFGL-NGS-21294	rs109138166	− 5.268	6.860	*EBF4*	Upstream	C/T
13	52,623,427	Hapmap47571-BTA-32877	rs41630007	− 5.91	8.466	*TMC2*	Intron	C/T
13	53,871,753	ARS-BFGL-NGS-82210	rs109449298	4.962	6.157	*PRPF6*	Upstream	T/C
13	54,192,189	ARS-BFGL-NGS-33209	rs109508313	− 4.962	6.157	*KCNQ2*	Intron	A/G
13	55,149,168	ARS-BFGL-NGS-104967	rs41697716	5.31	6.961	*TAF4*	Intron	A/G
13	55,687,392	Hapmap38200-BTA-120950	rs41624058	− 5.818	8.225	*CDH4-CDH26*	Intergenic	C/A
13	55,819,574	BTA-32981-no-rs	rs41630656	5.531	7.496	*CDH4-CDH26*	Intergenic	A/G
13	57,449,072	BTB-00531090	rs41694639	− 6.276	9.460	*NELFCD-GNAS*	Intergenic	C/A
13	57,988,686	Hapmap58840-rs29018856	rs29018856	− 5.287	6.904	*VAPB-RAB22A*	Intergenic	G/A
14	17,877,847	UA-IFASA-6459	rs42422981	− 5.767	8.092	*ENSBTAG00000043938-RF00026*	Intergenic	C/A
14	24,790,463	Hapmap32434-BTC-011497	rs41726059	− 4.904	6.026	*NSMAF*	Intron	C/T
14	26,212,648	Hapmap33173-BTC-073249	rs43054543	− 5.105	6.481	*RAB2A*	Intron	G/A
21	19,448,733	ARS-BFGL-BAC-33343	rs110246265	− 5.245	6.806	*RF00100-MRPL46*	Intergenic	G/A
29	28,560,818	ARS-BFGL-NGS-18412	rs110263608	5.588	7.640	*TMEM218-PKNOX2*	Intergenic	C/G

^1^Chr: Chromosome.

^2^iHS: Integrated Haplotype Score.

### Changes in EBV were detected in KPN cattle

**Figure 5:**
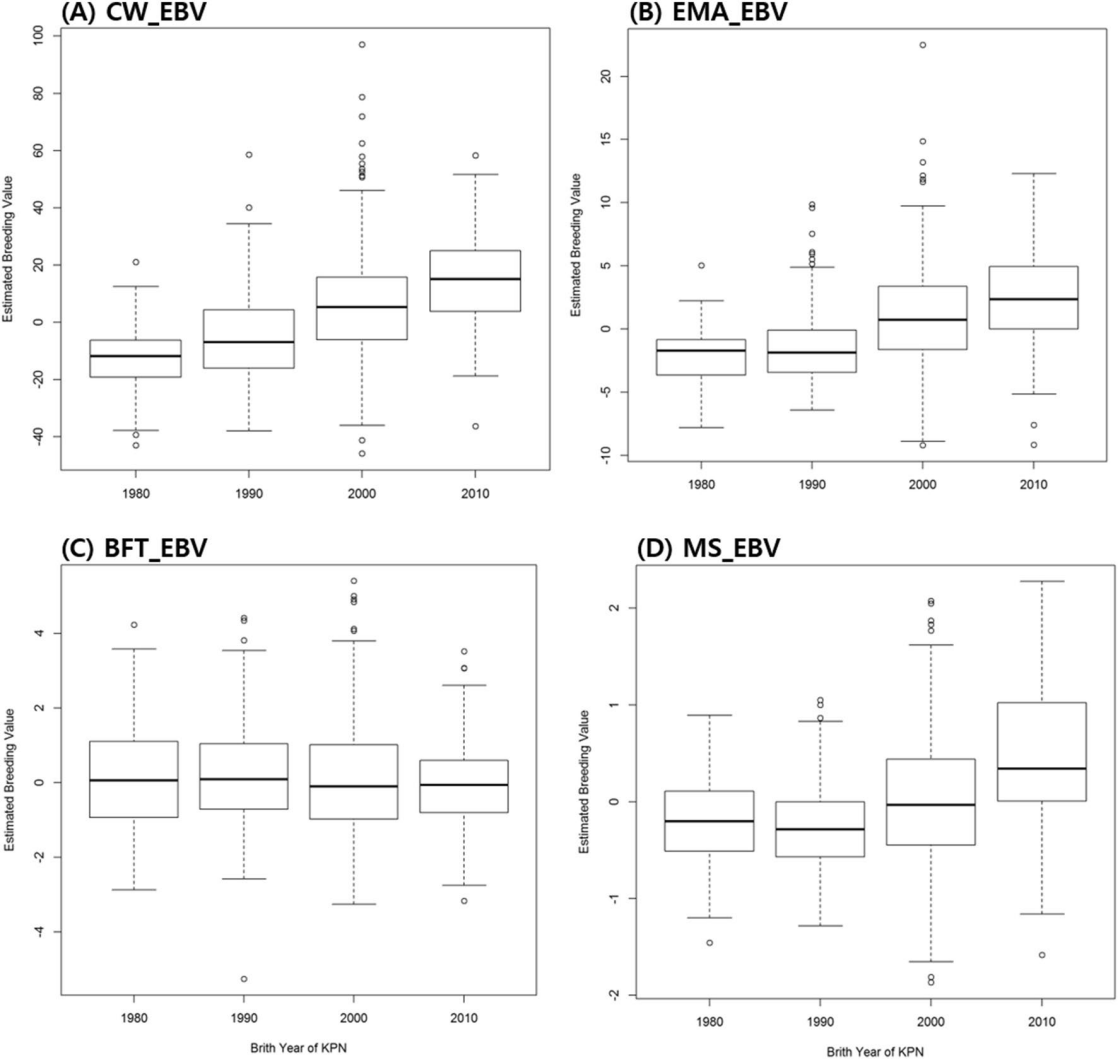
The estimated breeding value changes for each selected trait by decades. *EBV* estimated breeding value, *CW* carcass weight, *EMA* eye muscle area, *BFT* back fat thickness, *MS* marbling score.

**Figure 6:**
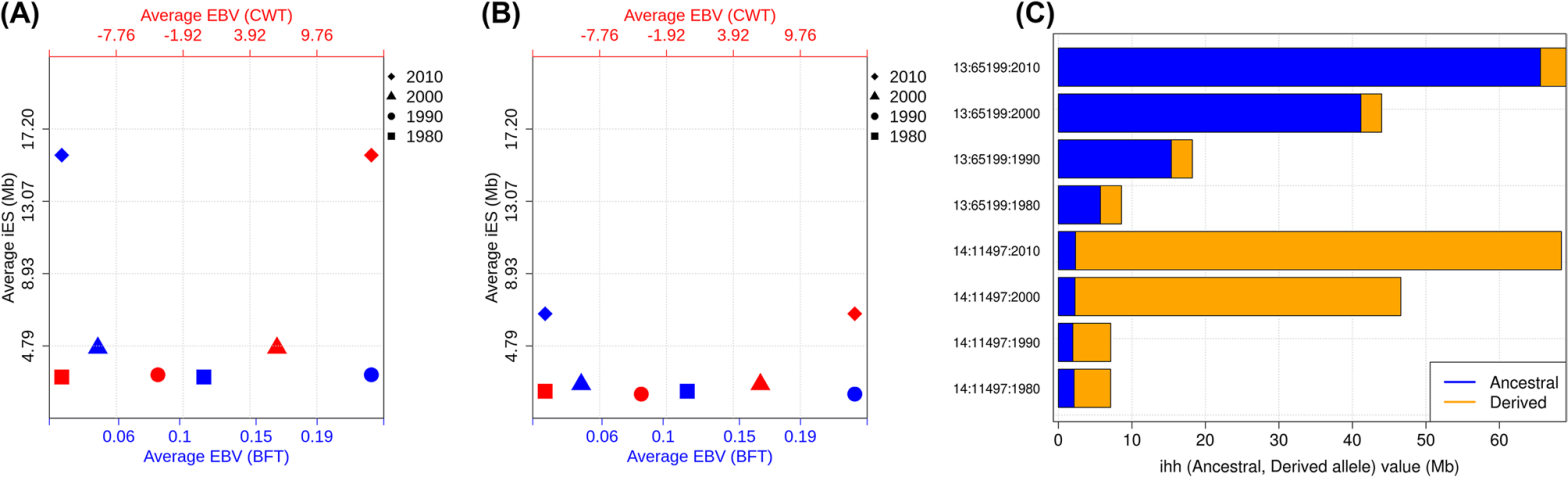
Haplotype increases according to EBV in 10-year increments identified in (**A**) ancestral and (**B**) derived alleles in KPN Hanwoo cattle selected for 0 years. (**C**) Decadal changes in the ratio of ancestral and derived haplotypes at ancestral and derived allele loci. In (**A**) and (**B**), the upper x-axis (red symbols) shows haplotype changes for CWT, and the lower x-axis (blue symbols) shows haplotype changes for BFT. An increase in haplotype (**C**) occurred in both alleles, and the ratios of ancestral and derived alleles differed.

The EBV values of the 667 KPN cattle selected from the 1980s to the 2010s were calculated and compared on a decadal basis. CWT (A), EMA (B), and MS (D) values increased continuously across these 10-year periods, whereas BFT (C) decreased slightly (Fig. 5A–D). Negative mean EBV values were observed in animals selected in the 1980s and 1990s, whereas later values were positive. The best linear unbiased prediction (BLUP) accounted for selection effects in the breeding population due to recently selected bulls showing higher selection effects caused by an accumulation of favorable alleles. CWT showed the highest selection differential among all examined traits, which can be explained by the significant genomic loci detected on BTA14 in the selection signature analysis (Fig. 5A). A comparison of EBV trends and haplotype expansion over 30 years confirmed that the haplotypes of ancestral and derived alleles expanded rapidly from the 2000s, with EBV becoming increasingly positive for CWT and increasingly negative for BFT (Fig. 6 and Fig. S5).

### GWAS of breeding traits in KPN cattle

**Figure 7:**
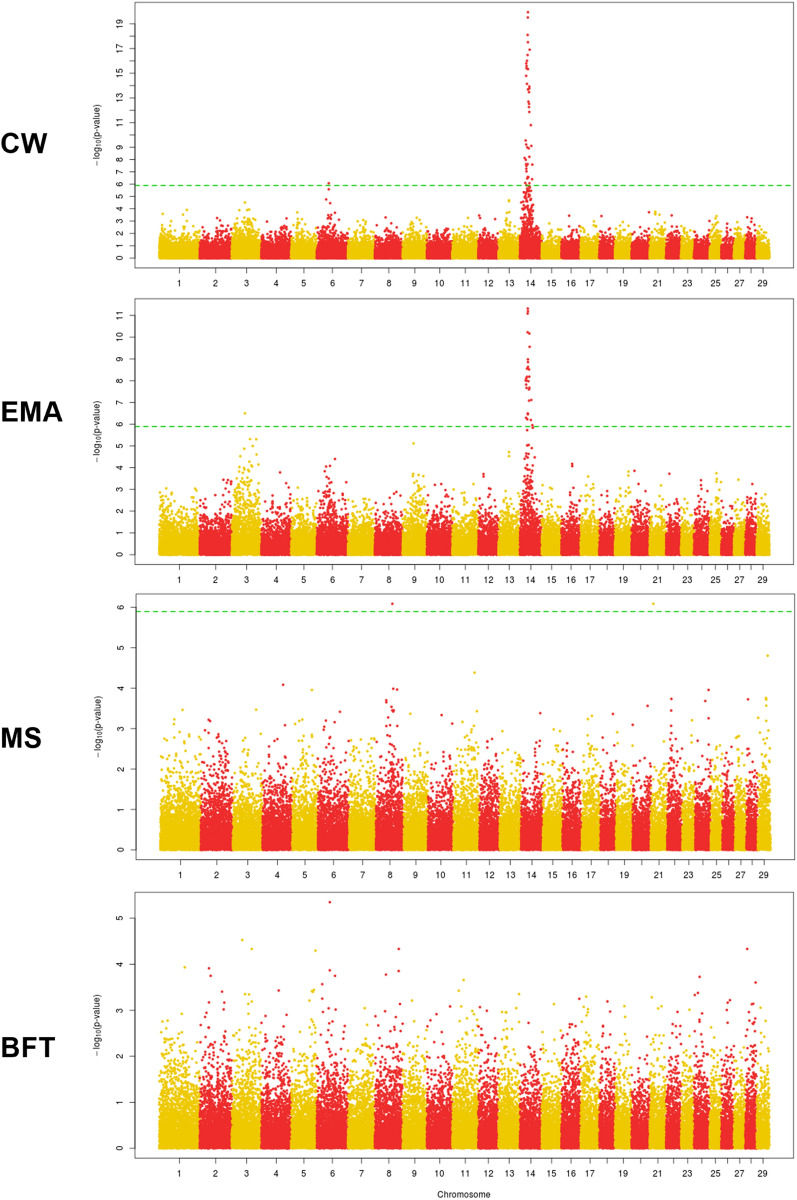
Genome-wide association study (GWAS) results for KPN cattle. *CWT* carcass weight, *EMA* eye muscle area, *BFT* back fat thickness, *MS* marbling score.

GWAS identified a significant genomic region for CWT on BTA14, adjacent to a region with a significant selection signature (Table S1, Fig. 7). Two significant genomic regions influencing CWT were identified on BTA6 (1 SNP) and BTA14 (44 SNPs). Among these, the genomic region on BTA14 spans from 15.5 to 44.9 M bp. Significant genomic regions influencing EMA, which showed high genetic correlation with CWT, were detected on BTA3 (1 SNP) and BTA14 (29 SNPs). The 29 SNPs showed a similar range (18–44 Mb) to that of CWT on BTA14. Only 1 SNP associated with MS was identified in each of BTA8 and BTA21. BFT-related SNPs were not identified in KPN (Table S1). A comparison of the selection signature analysis and GWAS results showed only one concordant genomic region on BTA14, whereas the iHS results showed four chromosomes (BTA13, 14, 21, and 29) harboring selection signature regions. Among these regions, one genomic region for CWT was detected on BTA14 in both analyses (Figs. 3 and 7). A significant genomic region for CWT was detected using GWAS on BTA6, whereas selection signature analysis did not detect this region, possibly because it was not fully segregated into the population. The other mismatched genomic region was BTA13, which showed a highly significant effect according to selection signature analysis but not according to GWAS.

### Functional annotation using KEGG and GO term analyses

GO and pathway analyses were performed for significant SNP positions on the BTA13 and BTA14 chromosomes. On BTA13, SNPs of the ancestral allele rs110693756 (ARS-BFGL-NGS-65199) and derived alleles rs110783267 (ARS-BFGL-NGS-14104) had the highest piHS scores. For GO analysis, we selected 19, 37, and 17 genes for each allele in the 1-Mbp regions to the right and left of the selected SNPs and searched the Ensemble Biomart database (https://asia.ensembl.org/info/data/biomart/index.html). In total, 127 functional genes were found in the ancestral allele, and 140 functional genes were found in the derived allele. Most of the ancestral alleles were related to DNA binding, ion binding in the cell, the nervous system, and digestive metabolism. Derived alleles were related mainly to digestive metabolism, fat, and animal behavior traits. To narrow down the range of functions for the list of genes, we identified functions with high frequency for BTA13 in the Cattle QTL Database (https://www.animalgenome.org/cgi-bin/QTLdb/BT/index); these were confirmed to be related mainly to energy metabolism and productivity. The gene list was re-filtered for major traits, resulting in 3 genes in the ancestral allele, and 16 and 3 genes in each derived allele for subsequent KEGG pathway analysis. No pathway was found for the ancestral allele. However, the derived allele rs110783267 (ARS-BFGL-NGS-14104) was identified in 45 pathways, of which 14 were significant, including those related to energy metabolism and efficiency, such as salivary secretion and the glucagon and insulin signaling pathways (Table S2, Fig. S6). The candidate genes associated with this pathway were found to be involved in four genes, *FOXA2*, *ACSS1*, *PYGB*, and *CALML5*. In BTA14, we performed GO analysis of the 56 genes in the 18–29 M bp region, based on the three significant SNPs identified through selection signature analysis. GWAS had already confirmed that BTA14 is associated with CWT and EMA, and *PLAG1* has a large effect size; therefore, we searched 4414 gene lists obtained through GO analysis for the *PLAG1* gene. In total, 30 pathways were identified among 345 functions among the resulting list of 29 genes (Table S3, Fig. S7). Of these, non-homologous end-joining and sulfur metabolism were identified as significant pathways, and the associated genes were identified as *IMPAD1* and *LYN*, respectively.

## Discussion

Cattle are among the largest populations of domesticated animals and used as food resources for humans; therefore, their phenotypes and genetic structure have been shaped by artificial selection for human needs and natural adaptation to environmental changes. The phenotypic selection causes genomic changes in breeding traits within breeds, resulting in altered genetic and phenotypic characteristics. In this study, we investigated changes in Hanwoo cattle genomic structure due to long-term artificial selection.

KPN cattle have been continuously selected for breeding traits during the past 30 years, whereas USP cattle have been maintained for conservation and research purposes. Therefore, these populations are expected to have different genetic structures in terms of haplotype and LD structure. However, an MDS plot showed little variation between KPN and USP cattle. Although the two populations are not genetically distinct groups, KPN cattle showed a clustered distribution, whereas USP cattle were widely and more uniformly distributed (Fig. S1). The exact reason for this result could not be determined using the genetic markers selected in the KPN cattle; however, we can infer a genetic difference between these populations by expanded haplotype sites and GWAS results. Although our admixture analysis results identified similar genetic components in the two populations, they were identified as different groups overall (Fig. 2). The results of our selection signature analysis showed between-population differences according to Rsb, whereas iHS showed more robust within-population differences (Fig. S8). The Rsb results showed that KPN and USP represent different selection signals. The haplotype of the positive selection was observed only in the KPN population through the iHS analysis results. The Rsb results show that the tendency is different from the selection signal in six chromosomes, including BTA13, which is estimated to be the genetic hitch-hiking effect on the selection traits of the strongly selected KPN population. The difference between MDS clustering and genetic components in the two groups is also assumed to be attributable to this genetic structure difference. The iHS results confirmed the results of the within-group selection signal based on the previously reported ancestral allele, detected haplotype for ancestral and derived allele in BTA13, and BTA14, a relatively recent positive selection signal compared to the BTA13. Strong selection on mutations associated with breeding traits can reduce variation around the region of causal mutation, forming specific haplotype sites^28,29^. Therefore, the lengths of such expanded haplotypes can be used as selection signatures^30^. Hanwoo cattle have undergone artificial selection for breeding traits, including CWT, EMA, MS, and BFT in a large-scale breeding program that relies on performance and progeny testing^3^.

In this study, we applied iHS to detect alleles of the selection signatures that have not yet reached fixation^31^ in KPN and USP cattle. Selection signatures were detected by identifying SNPs with positive or negative iHS values in KPN cattle but not in USP cattle. These results show that a strong selection of target traits in recent decades has been reflected in the changing genetic architecture of KPN cattle. On the other hand, USP cattle are not effective in the selection, so it is estimated that the genetic profile is relatively well preserved. In KPN cattle, iHS analysis showed that 8 ancestral and 29 derived alleles were detected, respectively, and that most haplotypes were recently fixed by selection (Table 1). Two genomic regions were identified as having been artificially selected in KPN cattle, with 25 SNPs from derived alleles and 7 SNPs from an ancestral allele on BTA13, and 3 SNPs for derived alleles on BTA14. Indeed, in the BTA14, the matching SNPs with previous studies for CWT and EMA traits were identified as derived alleles. However, GWAS did not show any association with the four breeding traits examined (CWT, EMA, BFT, and MS) on BTA13.

Selection signature analysis and GWAS both exploit LD to detect loci with causal mutations for breeding traits, and both have been used to analyze breeding traits in cattle^32,33^. In the present study, we detected strong selection signals on BTA13 and BTA14, although the genomic region of BTA13 showed no association with the four breeding traits examined in this study. One possible reason for this lack of association is that the selection signature of BTA13 is affected by the genetic hitch-hiking effects. These are highly correlated with selection traits (CWT, EMA, BFT, and MS) or due to essential survival traits that were selected together. For these reasons, we investigated BTA13 with other economic trait relationships in the cattle QTLdb. And we identified various association results for different productivity traits such as feed efficiency and reproduction traits^34,35^.

It is more likely that BTA13 is poorly annotated such that outlier SNPs are located in as-yet-undiscovered genes, but candidate genes within the significant genomic region from the genome-wide signature of selection and association analysis that are associated with milk yield and the interval from calving to first insemination (ICF) traits have been reported in this region. Among these, ICF is a complex trait that is affected by several physiological factors, and the identification of candidate genes is likely insufficient to determine its exact mechanism; however, since ICF is a determinant of early fertility performance and milk yield is an important factor in calf mammalian ability, both traits can affect the reproductive trait of cows. Based on these studies' results, we assume that other characteristics related to increased productivity (e.g., productivity, feed efficiency, and reproduction) have been selected during the Hanwoo cattle breeding program, which selected for only four specific traits. Our GO and pathway analysis results also identified candidate genes and pathways that may be associated with these productivity traits (Tables S2–S3). Among the 45 pathways identified for BTA13, the most significant pathways are related to energy metabolism and feed efficiency: glycolysis/gluconeogenesis, pyruvate metabolism, propanoate metabolism, starch, and sucrose metabolism, insulin signaling, glucagon signaling, and salivary secretion (Table S2, Fig. S6). Among these, five major genes, *FOXA2*, *ACSS1*, *PYGB*, *CALML5*, and *CST3*, play major roles in the identified pathways. The *FOXA2* (*Forkhead Box A2*) gene has a DNA binding function. This gene was identified as associated with the control of feeding behavior and energy homeostasis, including the better girl weight and body weight of Jianxian Red cattle, and Brahman cattle were reported reproduction trait relationships such as post-partum anoestrus interval (PPAI) and post-partum anoestrus interval with respect to weaning (PW)^36,37^. The *ACSS1* (*Acyl-CoA Synthesis Short-chain Family Member1*) gene functions in the conversion of acetate into Acyl-CoA and generation of ATP and CO_2_ through oxidation in the tricarboxylic acid cycle. These functions are involved in maintaining body temperature for energy homeostasis when the stomach is empty. In fact, Canovas et al.^38^ examined all metabolic phases of the citrate and fatty acid synthesis pathway in the ruminant mammary tissue to identify gene expression responsible for changes in citrate content in milk production and confirmed that the ACSS1 gene was involved in fatty acid synthesis and energy generation and NADPH regeneration. The *PYGB* (*Glycogen Phosphorylase B*) gene is a phosphorylase that regulates glycogen mobilization and is an important allosteric enzyme for carbohydrate metabolism. This gene has been reported as a gene that affects the tenderness and fat content of the Rectus abdominis muscle in cattle^39^. The *CST3* (*Cystain C*) gene is an inhibitor of cysteine proteinase and plays an important physiological role in controlling enzyme activity throughout the body. The major functions of the *CALML5* (*Calmodulin Like 5*) gene are calcium ion binding and encoding the calcium-binding protein associated with the calmodulin protein family; the resulting protein is a key enzyme in the final differentiation of keratinocytes. The functions of the reported genes are consistent with QTL associations and are directly or indirectly necessary to improve cow productivity.

Three significant SNPs identified in the selection signature analysis were associated with breeding traits (CWT and EMA) on BTA14. These SNPs are all derived alleles and are significant selection signals caused by strong artificial selection based on breeding values. SNPs associated with CWT have been reported for a 25-Mb region of BTA14 in Hanwoo cattle^40^, whereas in the present study, 44 SNPs were found to be significantly associated with CWT and EMA on BTA14 in KPN cattle due to strong selection during performance and progeny testing for CWT, EMA, BFT, and MS in the Hanwoo cattle breeding program. Performance testing selects candidate yearling bulls for their CWT and EMA properties^3,40^. This selection scheme has resulted in a dramatic increase in annual genetic gain for CWT and EMA in Hanwoo cattle^3^.

Also, BTA21 and BTA29 were found the selection signals but it did not detect a matching area of SNP in the GWAS analysis. Although the selection signal of BTA21 was identified as derived alleles in the 19.44 Mb region, the association result for MS in the BTA21 region from the GWAS results is somewhat distant, making it difficult to infer direct associations. When searching for the association information of the cattle QTLdb around this area, most of the features were found for Calving ease, Gestation length, Body weight, Udder depth, Milking speed, Milk kappa-casein percentage, and Stature, so the marbling relationship was not identified^41–43^. In this area, the ATP/GTP binding protein related *AGBL1* gene was identified in 17.5 Mb, and the *PEX11A* gene related to peroxisomal biogenesis in 21.1 Mb, possibly related to energy and fat metabolism. Adjacent genes in the 28.6 Mb region of BTA29 are found with genes related to the organic receptor family (*OR8* gene family) and the transforming growth factor (*TBRG1*), state and testis expression (*PATE* gene family), which are estimated to be related to the olfactory, reproduction, and growth function. These areas are selected by the genetic hitch-hiking effect, such as the selection signals of BTA13 regions, and further functional studies are needed in the future. Recent molecular and quantitative genetic studies have identified a causal mutation in the *PLAG1* zinc finger gene on BTA14; this mutation is strongly associated with CWT^44–46^. This gene is 52 k bp in size and located in the 25.00–25.05 M bp region of *Bos taurus UMD 3.1.1*. In this study, we detected no mutations in the *PLAG1* gene; however, the genomic region (rs41726059; Hapmap32434-BTC-011497*)* associated with CWT and EMA was located in adjacent regions of the *PLAG1* gene on BTA14. Of note, the results of our selection signature analysis showed a significant association with the rs41726059 (Hapmap32434-BTC-011497) SNP only in KPN cattle, not in USP cattle. Selection signature and GWAS analyses both detect loci associated with causal mutations based on LD within the population. Therefore, we investigated LD structure around significant SNPs on BTA14. We identified selection signatures in the rs41726059 (Hapmap32434-BTC-011497) and rs43054543 (Hapmap33173-BTC-073249) regions, due to their representative LD expansion locations (Fig. 4 and S9). In this LD structure, significant SNPs were located at both ends of an LD block that expanded to a size of 2.14 Mb; we estimate that the *PLAG1* gene located at the front of this block also forms an adjacent LD block (Fig. S10). Although no LD extension was observed among selection signature regions, this difference between the two populations is presumably due to changes in LD and haplotype structure caused by artificial selection. The decadal comparisons of EHH confirmed this expansion of the haplotype region, and the bifurcation analysis results show that one branch is decreasing. Thus, the ancestral and derived alleles show contradictory patterns. In the area identified with the derived allele, the number of branches has decreased, and a specific haplotype is prominently observed, whereas the ancestral allele has maintained the same number of branches. These changes in the haplotype branches are presumed to reflect differences between the ancestral allele, which has been preserved in the USP cattle, and the derived allele that has been altered through improvement of Hanwoo cattle.

Our decadal haplotype analysis showed that haplotype homozygosity increased every 10 years (Fig. 4). Due to prolonged artificial selection, haplotypes related to causal mutations have extended around significant SNPs. In this study, the ratio of ancestral and derived alleles in significant regions associated with CWT increased with EBV for the selection trait (Fig. 6), indicating that the genetic structure and haplotype changed due to selection pressure on the breeding target traits. These results clearly show that selection based on EBVs has been successful throughout the Hanwoo cattle breeding program. The recent implementation of genomic selection will likely accelerate these genetic responses during cattle selection.

## Conclusion

Strong selection can change genomic LD and haplotype structure following causal mutations among target traits. This study identified significant genome structure changes from ancestral to derived alleles due to selection on BTA13 and BTA14. In particular, the response of derived alleles shown in selection signatures in the BTA14 region provides evidence for their association with CWT, as reported in previous GWAS studies. Derived alleles in this region indicate expansion of the haplotype and LD blocks through an increase in selection signatures observed every 10 years. However, in the BTA13 region, where most selection signatures were detected, we found no association with the four selection traits; haplotype expansion was confirmed, but LD structural changes were not clearly identified, such that further association studies are needed using additional phenotype information. Notably, LD block structure changes were confirmed using a 50 k chip, which has low marker density and high average intervals between markers. Therefore, LD structural changes were confirmed only for the target trait, and detailed screening will be required for LD blocks using whole-genome sequence data. Hanwoo cattle have been subjected to intensive selection and productivity improvement during the past 30 years, and EBVs have clearly increased, resulting in the encoding of selection responses into genomic regions, even without the application of genomic selection. Therefore, genomic selection using this study results as basic data could accelerate Hanwoo cattle improvement as the first step in precision breeding to meet consumer demands.

## Supplementary Information

Supplementary Figure S1.

Supplementary Figure S2.

Supplementary Figure S3.

Supplementary Figure S4.

Supplementary Figure S5.

Supplementary Figure S6.

Supplementary Figure S7.

Supplementary Figure S8.

Supplementary Figure S9.

Supplementary Figure S10.

Supplementary Figure S11.

Supplementary Legends.

Supplementary Tables.
