# Supplementary figures and images for: Long-term artificial selection of Hanwoo (Korean) cattle left genetic signatures for the breeding traits and has altered the genomic structure
Source: Sci Rep. 2022 Apr 19;12:6438. doi: 10.1038/s41598-022-09425-0 (PMC9018707; doi:10.1038/s41598-022-09425-0)

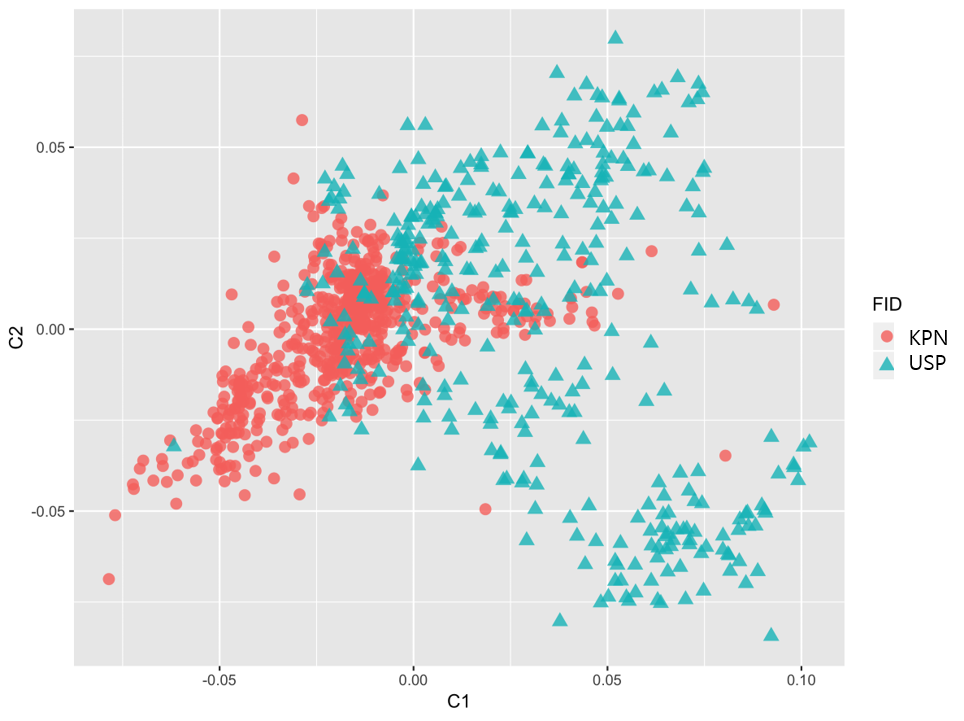

Supplement: Supplementary file 1 — Supplementary Figure S1. [file 41598_2022_9425_MOESM1_ESM.tif]

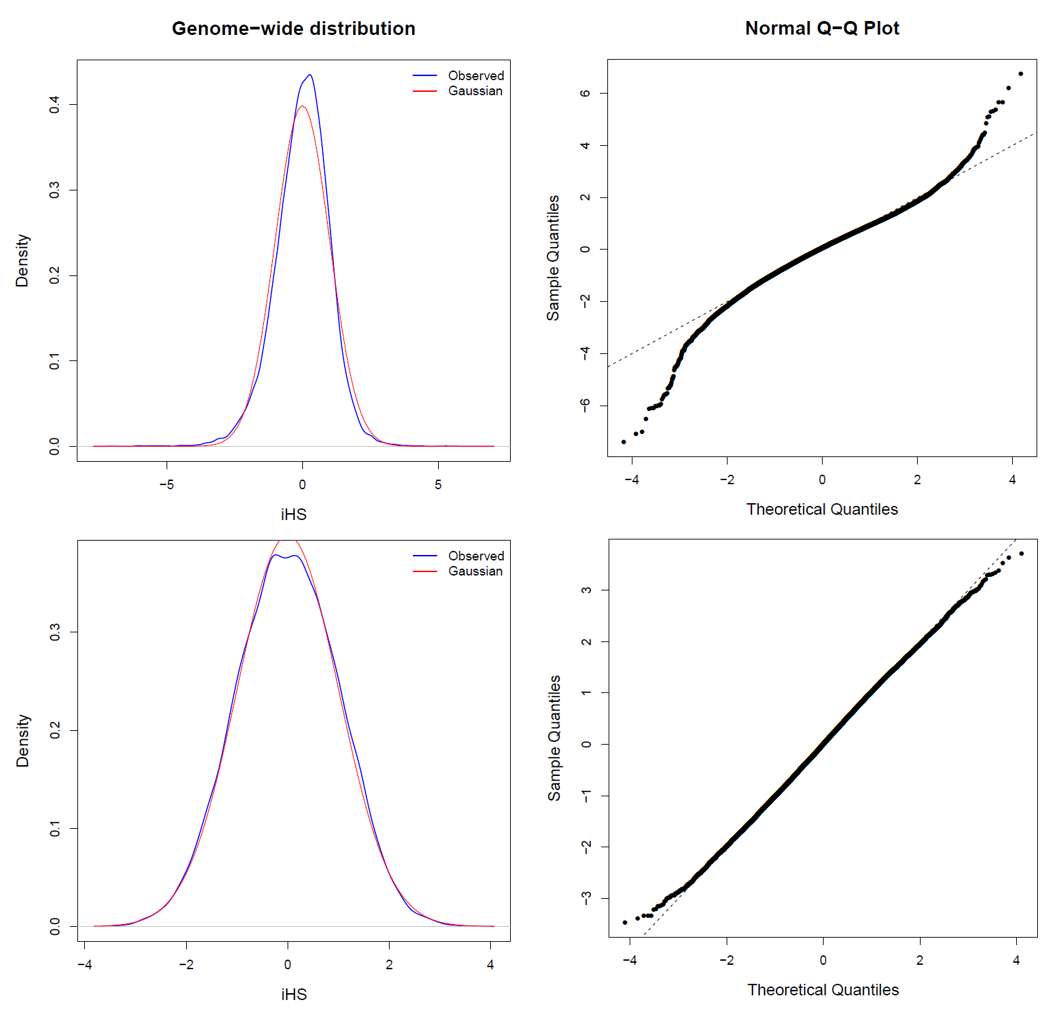

Supplement: Supplementary file 2 — Supplementary Figure S2. [file 41598_2022_9425_MOESM2_ESM.tif]

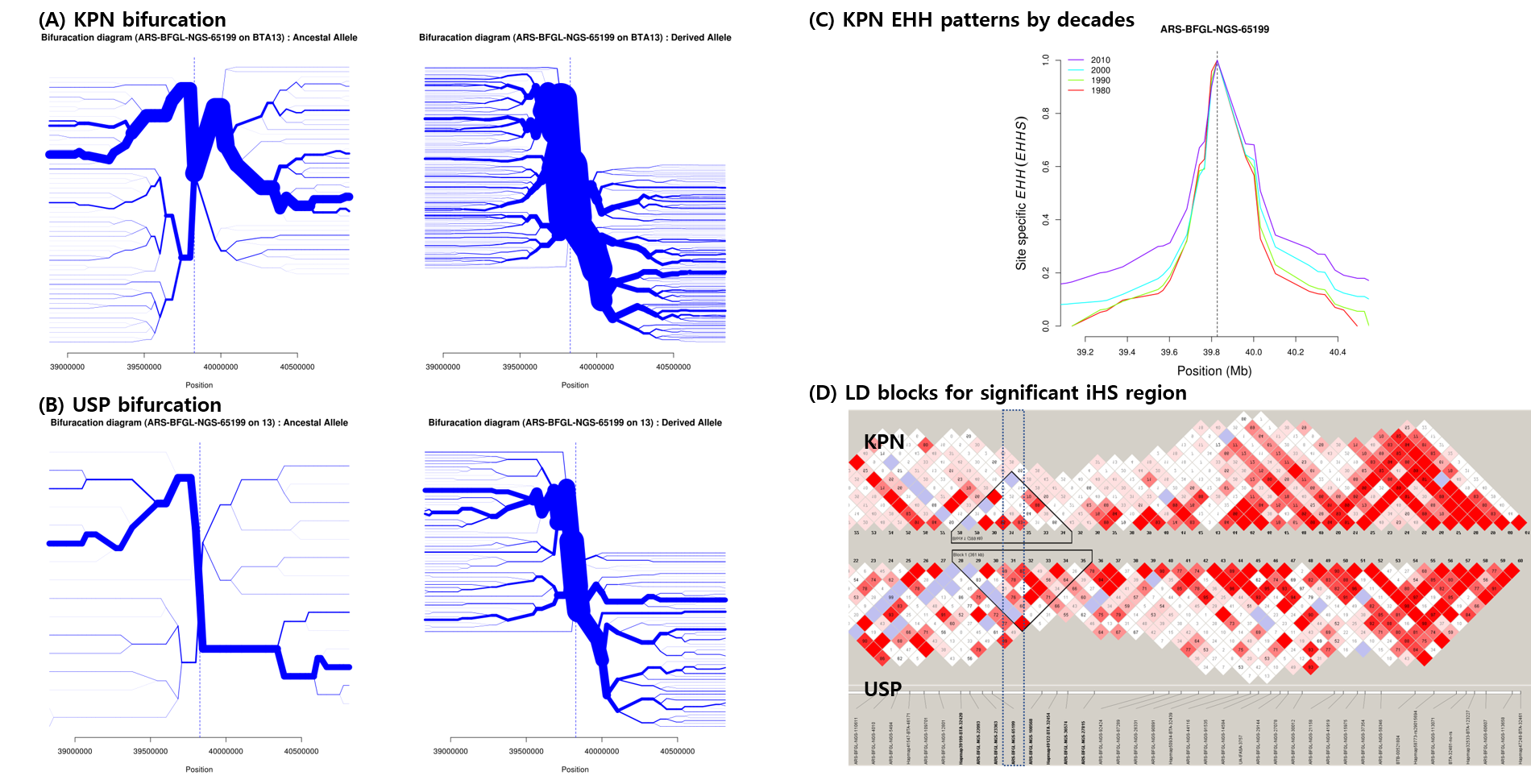

Supplement: Supplementary file 3 — Supplementary Figure S3. [file 41598_2022_9425_MOESM3_ESM.tif]

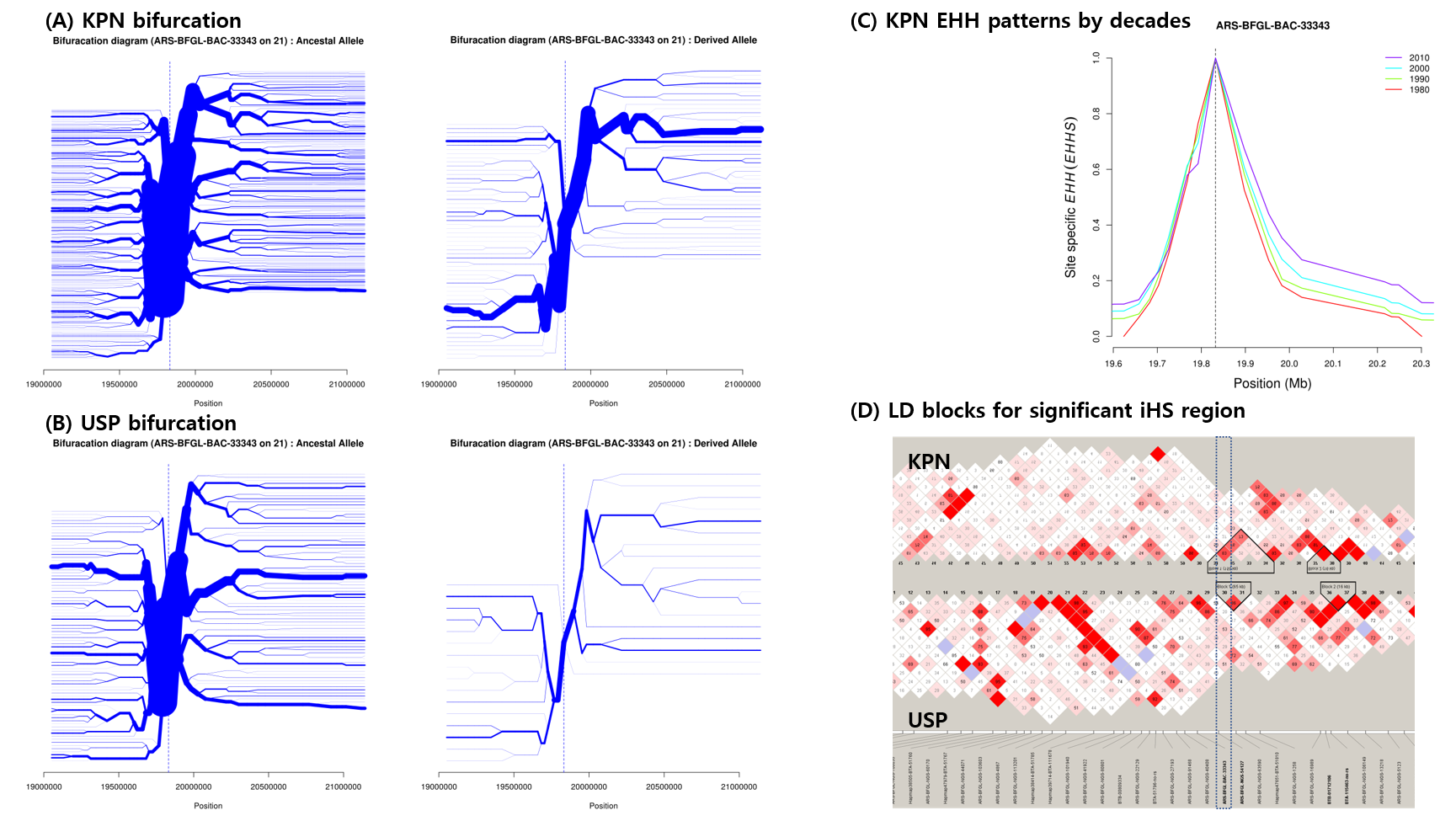

Supplement: Supplementary file 4 — Supplementary Figure S4. [file 41598_2022_9425_MOESM4_ESM.tif]

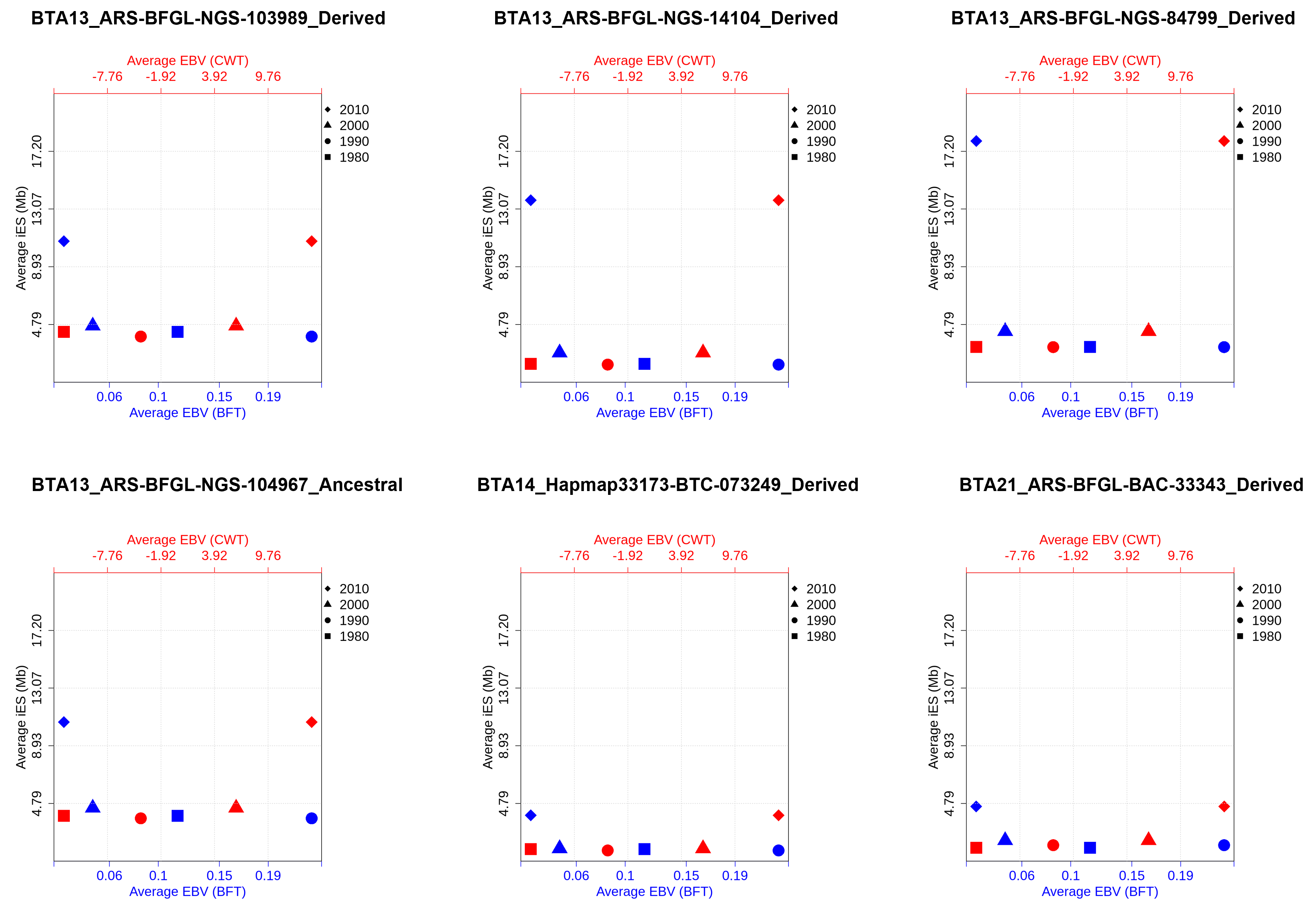

Supplement: Supplementary file 5 — Supplementary Figure S5. [file 41598_2022_9425_MOESM5_ESM.tif]

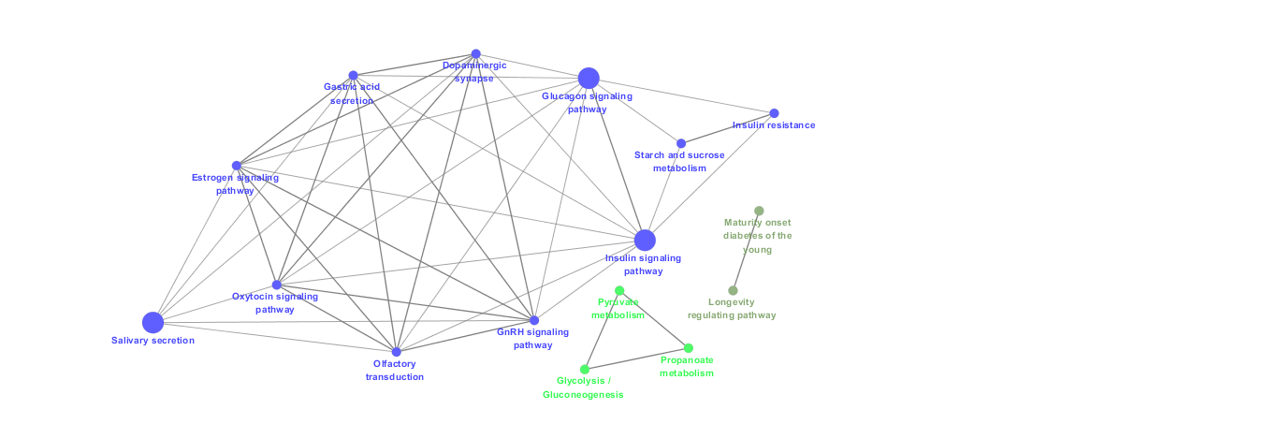

Supplement: Supplementary file 6 — Supplementary Figure S6. [file 41598_2022_9425_MOESM6_ESM.tif]

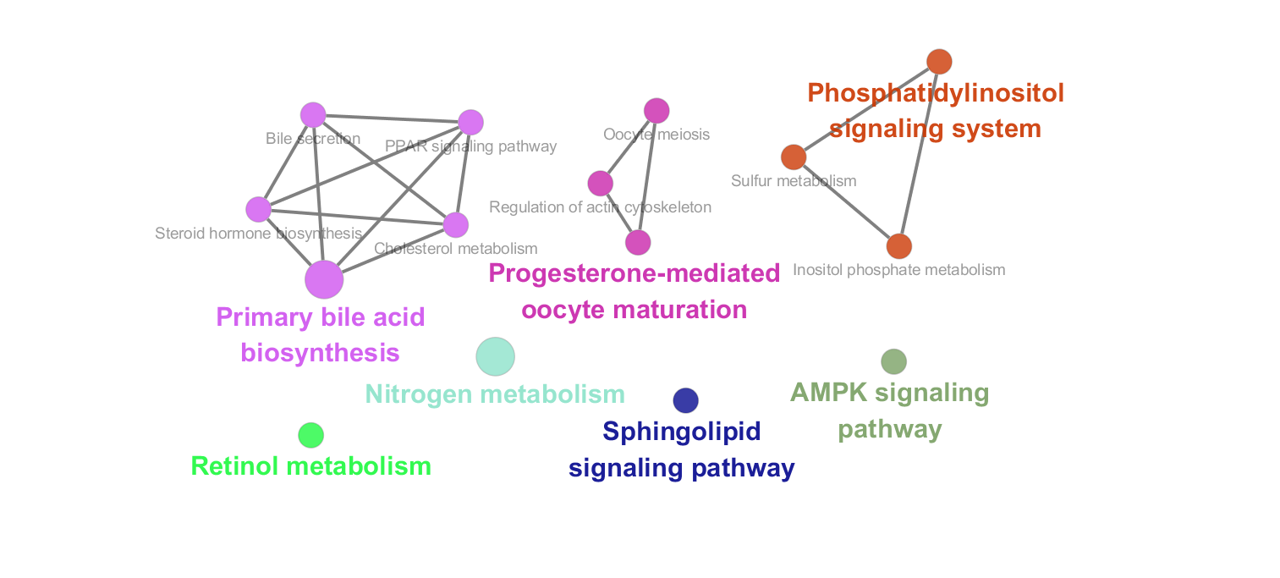

Supplement: Supplementary file 7 — Supplementary Figure S7. [file 41598_2022_9425_MOESM7_ESM.tif]

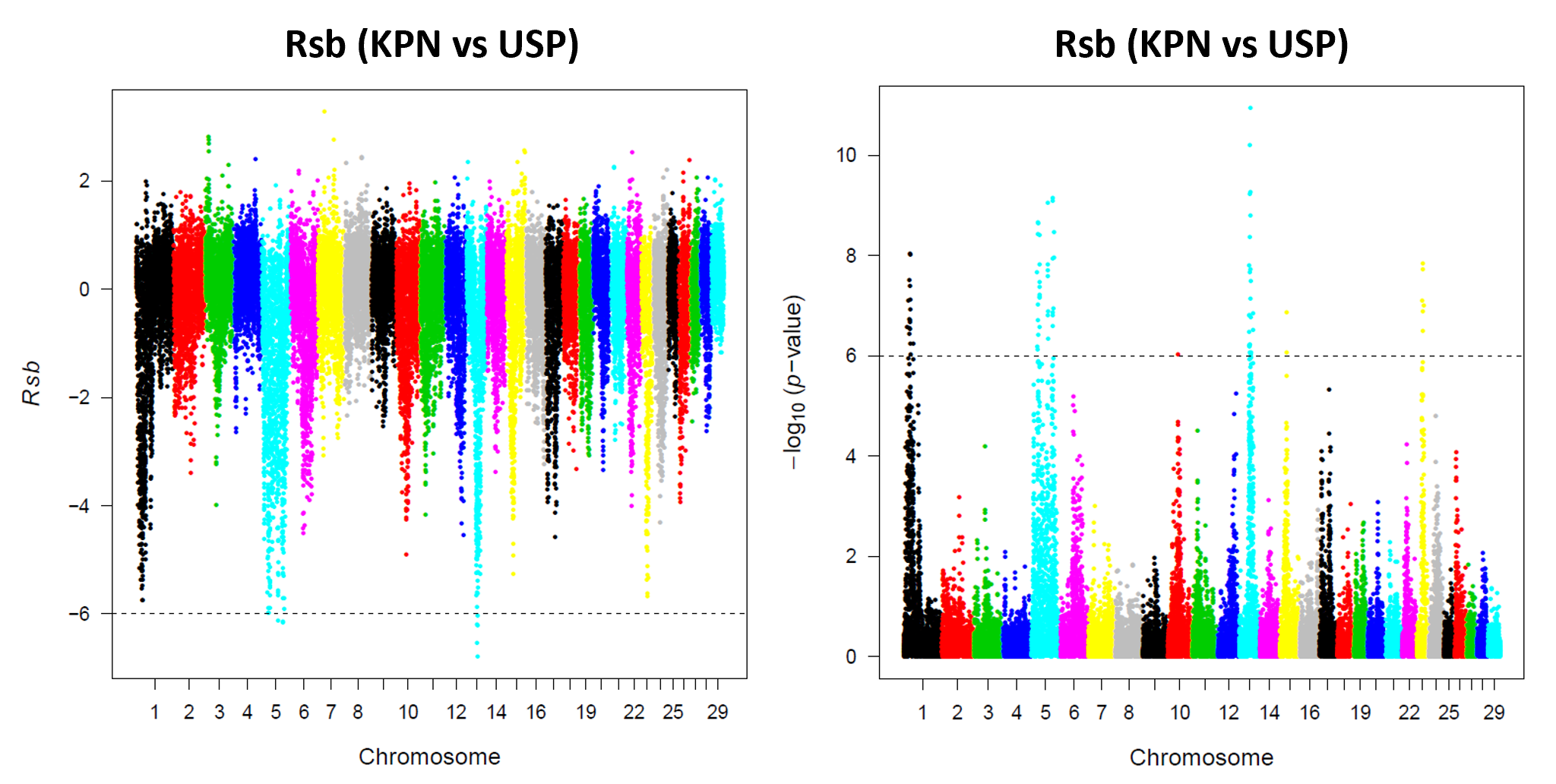

Supplement: Supplementary file 8 — Supplementary Figure S8. [file 41598_2022_9425_MOESM8_ESM.tif]

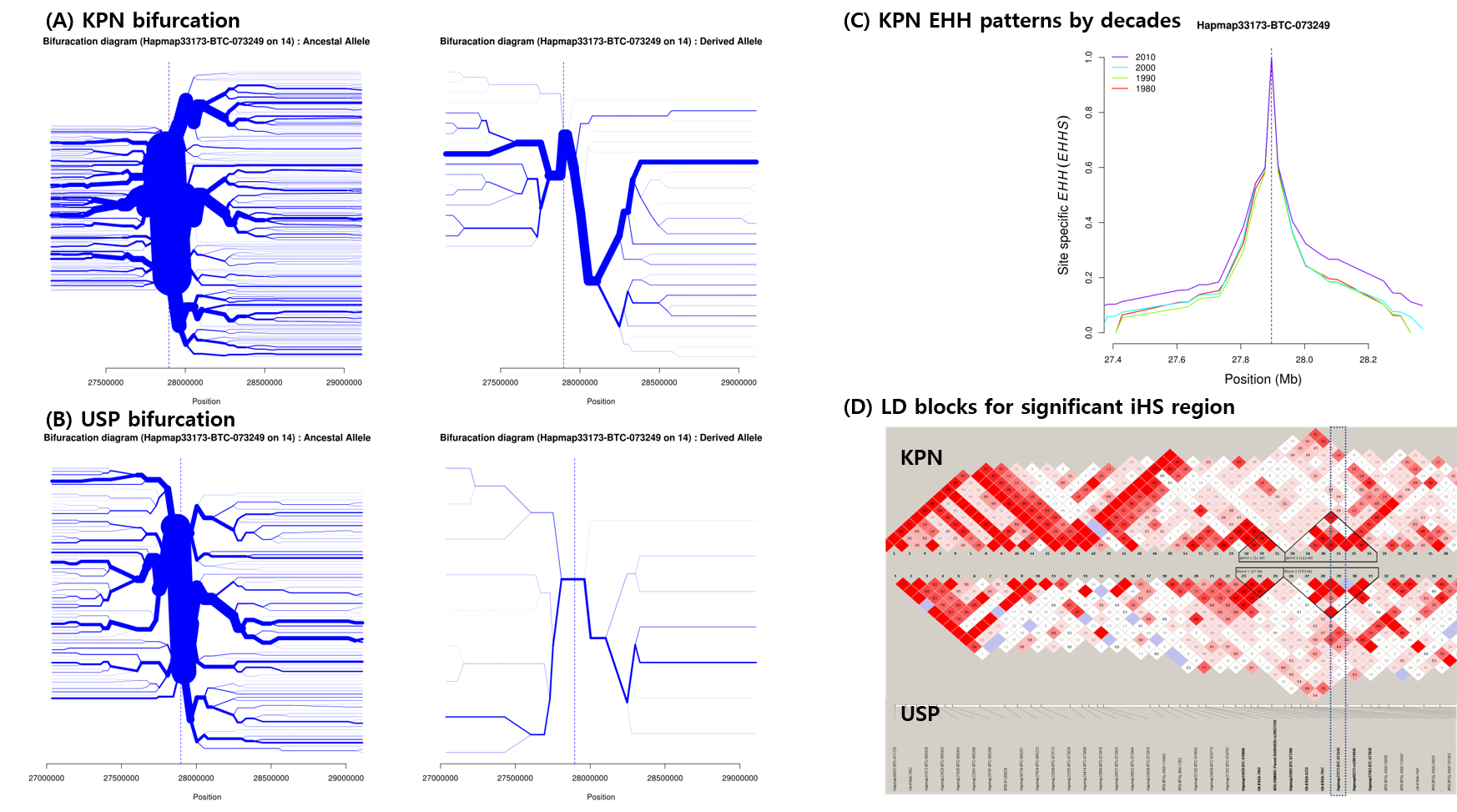

Supplement: Supplementary file 9 — Supplementary Figure S9. [file 41598_2022_9425_MOESM9_ESM.tif]

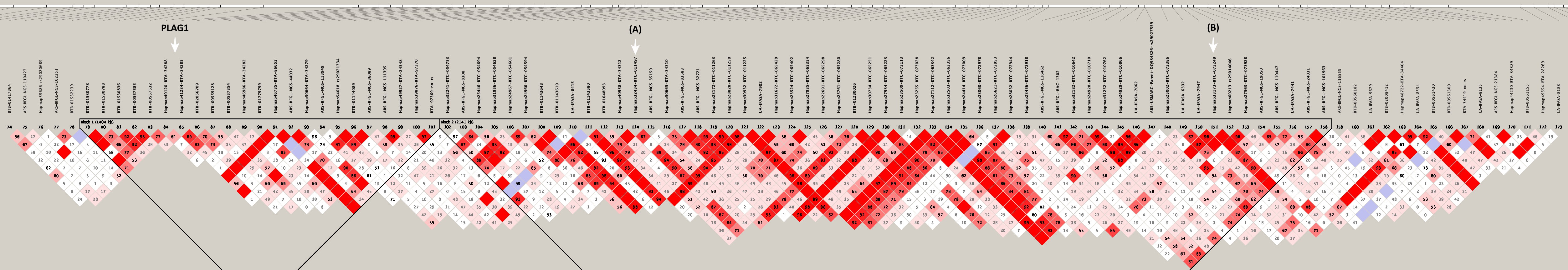

Supplement: Supplementary file 10 — Supplementary Figure S10. [file 41598_2022_9425_MOESM10_ESM.tiff]

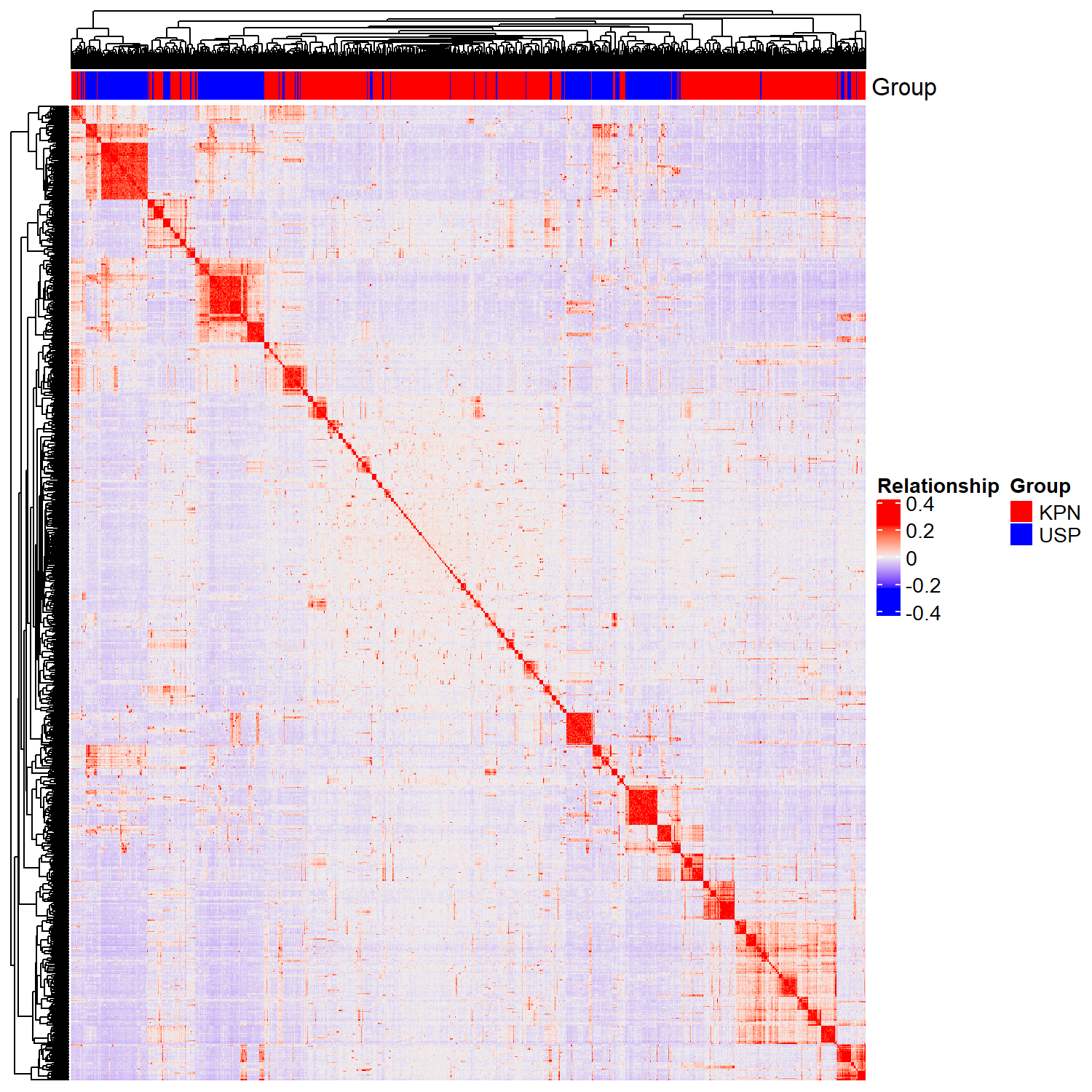

Supplement: Supplementary file 11 — Supplementary Figure S11. [file 41598_2022_9425_MOESM11_ESM.tiff]
