# Supplementary material for: Long-term artificial selection of Hanwoo (Korean) cattle left genetic signatures for the breeding traits and has altered the genomic structure
Source: Sci Rep. 2022 Apr 19;12:6438. doi: 10.1038/s41598-022-09425-0 (PMC9018707; doi:10.1038/s41598-022-09425-0)
Supplement: Supplementary file 12 — Supplementary Legends. [file 41598_2022_9425_MOESM12_ESM.docx]

**Legend of supplementary materials**

**Fig. S1. MDS plot selected (KPN) and unselected (USP) population:** The KPN clustered well than USP population and their distribution shared some of part.

**Fig. S2. Normalization, Gaussian distribution, and normal QQ plots show agreement of integrated haplotype score (iHS).**

**Fig. S3. The bifurcation, EHH and Haploview results for significant derived allele in Hanwoo:** (A) is bifurcation result of ancestral and derived allele for KPN population and (B) USP population. Both populations have strong ancestral haplotype in this significant associated region. (C) EHH result for KPN population were expanded more than 30 years ago and similar result with bifurcation and their (D) LD structure by Haploview analysis was also confirmed similar LD extension and structural change at ARS-BFGL-NGS14104 on BTA13.

EHH: expended haplotype homozygosity, KPN: 30-year selected Korean proven bull, USP: unselected population

**Fig. S4. The bifurcation, EHH and Haploview results for significant derived allele in Hanwoo:** (A) is bifurcation result of ancestral and derived allele for KPN population and (B) USP population. Both populations have strong ancestral haplotype in this significant associated region. (C) EHH result for KPN population were expanded more than 30 years ago and similar result with bifurcation and their (D) LD structure by Haploview analysis was also confirmed similar LD extension and structural change at ARS-BFGL-BAC-33343 on BTA21.

EHH: expended haplotype homozygosity, KPN: 30-year selected Korean proven bull, USP: unselected population

**Fig. S5. The trend of haplotype increases for EBV in 10-year increments identified in significant derived and ancestral allele of KPN Hanwoo selected for 30 years.** The upper x-axis and red symbols represent haplotype change for carcass weight (CWT), and the lower x-axis and blue symbols represent haplotype change for back fat thickness (BFT). In both alleles, an increase in haplotype occurred (C) and the ratios of ancestral and derived alleles were different.

EBV: estimated breeding value, CWT: carcass weight, BFT: back fat thickness

**Fig. S6. Gene ontology (GO) and Kyoto Encyclopedia of Genes and Genomes (KEGG) pathway analysis results for significant selection signature regions on BTA13.** In this area, we mainly detected genes related to salivary secretion, gastric acid secretion, glucagon and insulin pathways related to nutrient physiology, and estrogen and oxytocin signaling pathways related to reproduction.

**Fig. S7. GO and KEGG pathway analysis results for significant selection signature regions on BTA14.** Notably, sulfur metabolism directly affects protein synthesis and production function, which are related to CWT. The non-significant AMPK signaling, and folate biosynthesis pathways are involved in immunity, growth, and metabolism, which are also related to CWT.

**Fig. S8. Analyses of Rsb values to identify differences between the selected (KPN) and unselected (USP) Hanwoo populations.**

**Fig. S9. The bifurcation, EHH and Haploview results for significant derived allele in Hanwoo:** (A) is bifurcation result of ancestral and derived allele for KPN population and (B) USP population. Both populations have strong ancestral haplotype in this significant associated region. (C) EHH result for KPN population were expanded more than 30 years ago and similar result with bifurcation and their (D) LD structure by Haploview analysis was also confirmed similar LD extension and structural change at Hapmat33173-BTC-073249 on BTA14.

EHH: expended haplotype homozygosity, KPN: 30-year selected Korean proven bull, USP: unselected population

**Fig. S10. The location of the *PLAG1* gene and the LD expansion of the two SOS significant derived SNPs.** (A) is rs41726059 and (B) is rs43054543, respectively. Two SNPs have been expanded as 2.1 M bp block size and the *PLAG1* gene also confirmed the trend of expansion.

**Fig. S11. The heatmap result by genetic relationship matrices (GRM) for evaluation of individual genetic distances by GCTA software**

**Table S1. GWAS results for KPN population**

**Table S2. KEGG pathway results for BTA13**

**Table S3. KEGG pathway results for BTA14**

**Table S4. List of significant single-nucleotide polymorphisms (SNPs) from the integrated haplotype score (iHS) results by threshold 3 in KPN Hanwoo group**

**Table S5. List of significant single-nucleotide polymorphisms (SNPs) from the integrated haplotype score (iHS) results by threshold 3 in USP Hanwoo group**
